# Adenosine as an Adjunctive Therapy for Acute Myocardial Infarction Undergoing Primary Percutaneous Coronary Intervention: A Systematic Review and Meta-Analysis of Randomized Controlled Trials

**DOI:** 10.31083/RCM24065

**Published:** 2025-02-12

**Authors:** Xue-Mei Feng, Wen-Hui Zhang, Jia Liu

**Affiliations:** ^1^School of Basic Medical Sciences, Shanghai Jiaotong University, 200025 Shanghai, China; ^2^Department of Digestive Oncology, Baotou Cancer Hospital, 014030 Baotou, Inner Mongolia, China; ^3^School of International Pharmaceutical Business China Pharmaceutical University, 210009 Nanjing, Jiangsu, China

**Keywords:** adenosine, acute myocardial infarction, percutaneous coronary intervention, cardioprotection, no-reflow, infarct size, reperfusion injury

## Abstract

**Background:**

Adenosine administration can improve coronary blood flow in patients undergoing primary percutaneous coronary intervention (PCI); however, the therapeutic effects of adenosine on ST resolution and major adverse cardiovascular events (MACEs) after PCI remain unclear. This study aimed to assess the therapeutic effects of adjunctive adenosine administration on patients with acute myocardial infarction (AMI) undergoing PCI using a meta-analytic approach.

**Methods:**

We conducted a systematic search across PubMed, Embase, and the Cochrane Library to identify eligible randomized controlled trials (RCTs) published from inception through to March 2024. Primary outcomes included ST resolution and MACEs. The pooled analyses were all conducted using the random-effects model. Additionally, exploratory analyses were carried out through the application of sensitivity and subgroup analyses.

**Results:**

Twenty-one RCTs involving 2467 patients with AMI were selected for the meta-analysis. Adenosine significantly increased the incidence of ST resolution (relative risk [RR]: 1.30; 95% confidence interval [CI]: 1.15–1.46; *p* < 0.001), while it significantly reduced the risk of MACEs (RR: 0.67; 95% CI: 0.51–0.87; *p* = 0.003). Moreover, the use of adenosine was associated with reduced incidences of no reflow (RR: 0.35; 95% CI: 0.24–0.52; *p* < 0.001) and myocardial blush grade (MBG) 0 to 1 (RR: 0.75; 95% CI: 0.58–0.99; *p* = 0.041). Furthermore, adenosine significantly reduced the risk of heart failure (RR: 0.66; 95% CI: 0.44–0.99; *p* = 0.044). Finally, adenosine use was associated with a lower creatine kinase-MB (CK-MB) peak value (weighted mean difference: –36.94; 95% CI: –73.76– –0.11; *p* = 0.049).

**Conclusions:**

This study revealed that adenosine use was associated with an increased incidence of ST resolution, and reduced risk of MACEs.

**The INPLASY registration:**

INPLASY202510051, https://inplasy.com/inplasy-2025-1-0051/.

## 1. Introduction

Cardiovascular disease (CVD) has contributed to a major burden on global health, and deaths related to CVD have increased during the past three decades [1]. Cardiac mortality is predominantly attributed to coronary artery disease, notably acute myocardial infarction (AMI), and the ensuing complications. AMI arises from severe and prolonged ischemia or necrosis of the myocardium, with resultant clinical complications such as heart failure, cardiogenic shock, arrhythmias, cardiac arrest, and mechanical issues affecting the heart’s function [2, 3]. Platelet adherence, activation, and aggregation on the injured thrombotic surface, triggered by coronary plaque fissures, erosions, or ruptures entering the bloodstream, are intimately tied to the advancement of thrombotic processes and lead to vascular stenosis or blockage. Therefore, the ischemic myocardium can be improved by restoring blocked coronary blood flow after AMI [4].

Currently, emergency or elective percutaneous coronary intervention (PCI) is widely used for revascularization in patients with AMI [5]. The severity of ischemic injury and cardiac function could improve in patients who receive early reperfusion by primary PCI, and the mortality risk is significantly reduced [6, 7, 8]. However, the prevalence of the no reflow phenomenon for patients treated with primary PCI remains high (range, 5%–50%) and is associated with poor clinical outcomes and mortality; therefore, additional preventive strategies should be identified [7, 9, 10]. Because the no reflow phenomenon has a multidimensional pathophysiology, various strategies have been introduced in clinical practice for primary PCI, including preprocedural medication and intracoronary agents [11, 12, 13].

Adenosine is an endogenous purine nucleoside that can inhibit neutrophil activation and platelet aggregation, prevent endothelial damage, and dilate the coronary vessels, which is widely used in clinical practice to prevent and improve the no reflow or slow reflow phenomenon. The no-reflow phenomenon typically occurs at the microvascular level, where even after successful reperfusion of larger vessels, microvascular damage and spasm can still restrict blood flow. Adenosine, by directly dilating microvessels, alleviates microcirculatory impediments, facilitating effective reperfusion of myocardial cells, and thereby reducing the incidence of no-reflow or slow-reflow [14, 15]. Numerous studies have investigated the efficacy and safety of intravenous and intracoronary administration of adenosine for patients with AMI undergoing PCI. However, these studies reported inconsistent results because of their various routes, doses, and methods of detecting no reflow. Therefore, the current systematic review and meta-analysis was performed to update the efficacy and safety of adenosine for patients with AMI undergoing PCI. Moreover, exploratory analyses were also performed to explore any potential therapeutic effects of adenosine for patients with AMI undergoing PCI.

## 2. Methods

### 2.1 Data Sources, Search Strategy, and Selection Criteria

This systematic review and meta-analysis were conducted according to the guidelines outlined in the Preferred Reporting Items for Systematic Reviews and Meta-Analyses Statement [16]. Eligibility for inclusion in our study were randomized controlled trials (RCTs) examining the use of intravenous or intracoronary adenosine administration in AMI patients undergoing PCI, with no limitations imposed on the publication language or status. PubMed, Embase, and the Cochrane Library were systematically searched for eligible RCTs published through March 2024. The search terms used were (“adenosine”) AND (“primary percutaneous coronary intervention” OR “ST elevation myocardial infarction” OR “primary PCI” OR “acute myocardial infarction” OR “no Reflow”). We searched the websites of ClinicalTrials.gov (United States National Institutes of Health) to identify trials that had been completed but not yet published. Furthermore, the reference lists of the original and review articles were manually reviewed to identify additional studies that met the criteria. Study selection was performed based on the medical subject heading, study design, patient population, intervention, control, and outcome variables.

Two reviewers independently carried out the literature search and study selection employing consistent methodology. Any disagreements between the reviewers were resolved through team discussions until a mutual agreement was achieved. The criteria for including studies in our analysis were as follows: (1) patients: AMI and undergoing PCI; (2) intervention: intravenous or intracoronary administration of adenosine; (3) control: placebo as the control; (4) outcomes: the primary outcomes including ST resolution and major adverse cardiovascular events (MACEs), while the secondary outcomes including no reflow, myocardial blush grade (MBG) 0 to 1, all-cause mortality, cardiac death, thrombosis, reinfarction, heart failure, advanced atrioventricular (AV) blocks, hypotension, ventricular tachycardia (VT)/ventricular fibrillation (VF), bradycardia, creatine kinase-myocardial band (CK-MB) peak value, and left ventricular ejection fraction (LVEF); (5) study design: the study had to have an RCT design.

### 2.2 Data Collection and Quality Assessment

The information extracted from the eligible RCTs included the first author’s surname, publication year, country, sample size, age, male proportion, hypertension proportion, proportion with diabetes mellitus (DM), proportion of smokers, setting, definition of ST resolution, route of adenosine, intervention, ischemic time to therapy, outcome definition (MACE definition), follow-up duration, and reported outcomes. The quality of the methodologies employed in the included trials was evaluated utilizing the Cochrane Risk of Bias tool [17]. Data extraction and evaluation of study quality were executed separately by two reviewers. In instances where discrepancies arose, a third reviewer was consulted to resolve the disagreement through a reference to the primary source material.

### 2.3 Statistical Analysis

The therapeutic effects of adenosine compared to placebo were quantified as relative risks (RR) for categorical outcomes and weighted mean differences (WMD) for continuous outcomes, each accompanied by a 95% confidence interval (CI). A random-effects model was employed in the calculation of the combined effect size to account for the inherent heterogeneity among the included studies [18, 19]. Variability or heterogeneity among the included trials was examined using the *I*^2^ statistic and the Q test. Heterogeneity was deemed substantial when the *I*^2^ value exceeded 50.0% or the *p*-value was less than 0.10 [20, 21]. The stability of the combined findings was tested by conducting a sensitivity analysis, which involved iteratively excluding individual trials from the analysis to ascertain the consistency and reliability of the overall conclusion [22]. Subgroup analyses were performed for ST resolution and MACEs based on age, male sex, hypertension, DM, current smoking, route of adenosine administration, and ischemic time to therapy; furthermore, the differences between subgroups were compared using the interaction *t*-test, which assumes that the data were normally distributed [23]. Publication bias for ST resolution and MACEs was assessed using funnel plots, Egger tests, and Begg tests [24, 25]. All reported *p*-values were two-sided, and statistical significance was defined as *p* < 0.05 for pooled conclusions. All analyses were conducted using STATA software (version 14.0; Stata Corporation, College Station, TX, USA).

## 3. Results

### 3.1 Literature Search

**Fig. 1.**
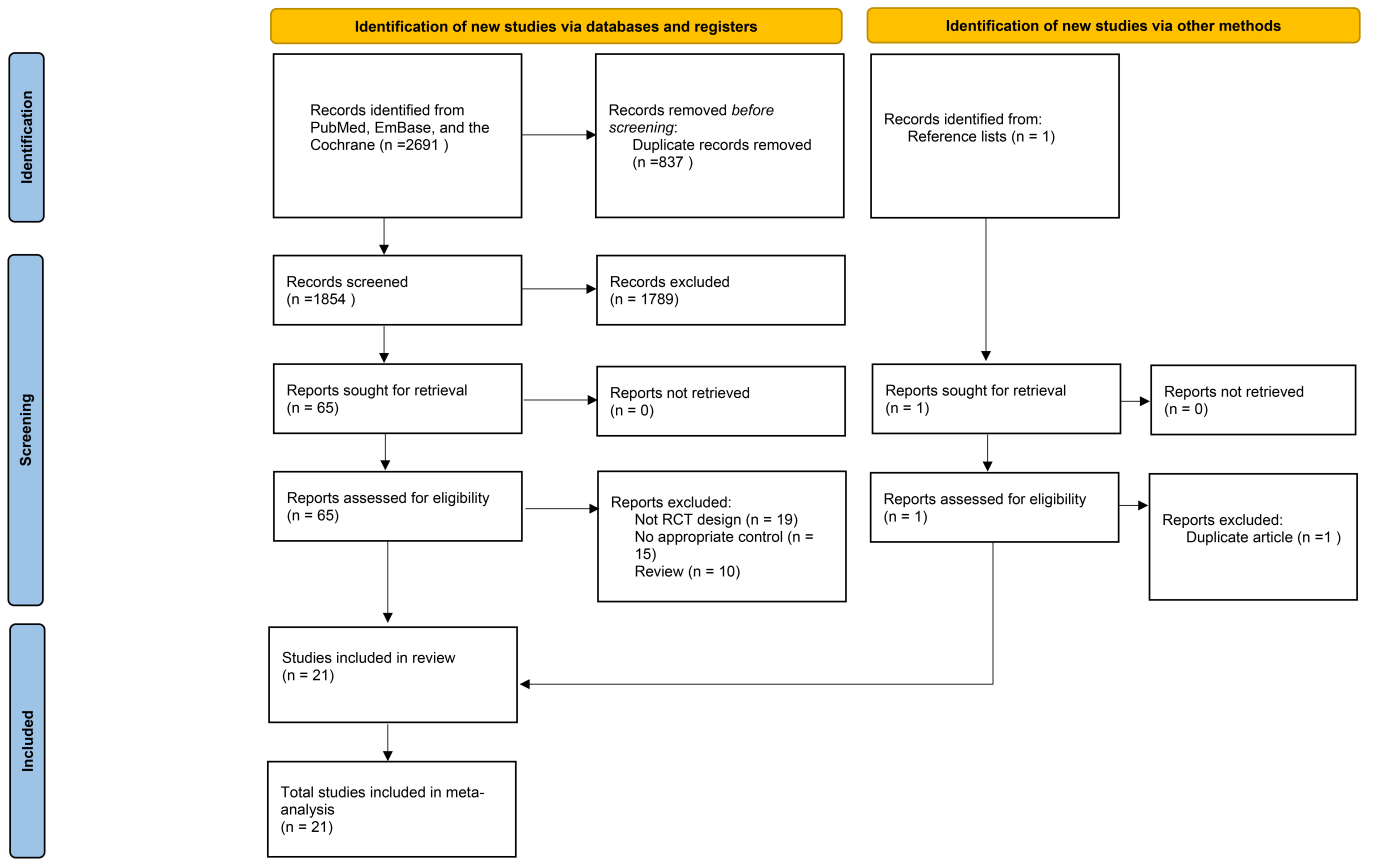
**The PRISMA flowchart regarding the details of the literature search and study selection**. RCTs, randomized controlled trials.

Fig. 1 illustrates the literature search and study selection processes. Initially, our electronic search retrieved 2691 records, which were reduced to 1854 entries after eliminating duplicates. Following this, 1789 of these articles were discarded due to their irrelevance to the research topic. Ultimately, 65 studies were chosen for comprehensive full-text assessment. Subsequently, 44 studies were excluded because they did not include an RCT design (n = 19), appropriate control (n = 15), or review (n = 10). A review of the reference lists did not identify new eligible trials that met the inclusion criteria. Finally, 21 RCTs were selected for the final quantitative analysis [26, 27, 28, 29, 30, 31, 32, 33, 34, 35, 36, 37, 38, 39, 40, 41, 42, 43, 44, 45, 46].

### 3.2 Study Characteristics 

The baseline characteristics of identified studies are shown in Table 1 (Ref. [26, 27, 28, 29, 30, 31, 32, 33, 34, 35, 36, 37, 38, 39, 40, 41, 42, 43, 44, 45, 46]). In the 21 included trials, 2467 patients with AMI were included, and the follow-up duration ranged from in-hospital to 12.0 months. 20 trials included patients with ST-elevation myocardial infarction (STEMI) [26, 27, 28, 29, 31, 32, 33, 34, 35, 36, 37, 38, 39, 40, 41, 42, 43, 44, 45, 46], whereas the remaining 1 trial included both STEMI and non-STEMI (NSTEMI) [30]. 18 trials used intracoronary adenosine administration [26, 27, 29, 30, 31, 32, 33, 34, 35, 36, 37, 40, 41, 42, 43, 44, 45, 46], and the remaining 3 trials used intravenous adenosine administration [28, 38, 39]. **Supplementary Table 1** summarizes the methodological quality of each trial, and the overall quality was moderate.

**Table 1.** **Baseline characteristics of eligible trials and involved patients**.

Study	Country	Sample size	Age (years)	Male (%)	Hypertension (%)	DM (%)	Smoking (%)	Setting	STR (%)	Stent usage (%)	TIMI flow	Intervention	Ischemic time to therapy	Outcome definition	Follow-up duration
Marzilli 2000 [26]	Italy	54 (27/27)	60.2	79.6	NA	NA	NA	STEMI	NA	16.7	NA	The balloon was inflated, and adenosine (IC, 4 mg in 1 min) was hand-injected into the distal vascular bed	116 min	No-reflow was diagnosed when a reduction of ≥1 TIMI grades; MACE: recurrent angina/ischemic, nonfatal AMI, cardiac death	In-hospital
Claeys 2004 [27]	Belgium	279 (79/200)	61.1	76.6	43.4	11.8	47.7	STEMI	50	80.3	NA	Adenosine (IC, 60 µg/min for RCA and 90 µg /min for LCA) was administered just before and during PCI after 20-min	247 min	MACE: nonfatal AMI and cardiac deaths	1.0 month
Micari 2005 [28]	USA	30 (14/16)	57.0	66.7	53.3	20.0	NA	STEMI	NA	100.0	NA	Adenosine (IV, 50–70 µg/kg/min for 3 hours), initiated <15 min before the procedure	292 min	—	1.0 month
Petronio 2005 [29]	Italy	60 (30/30)	58.5	85.0	NA	20.0	50.0	STEMI	50	100.0	0–1	Adenosine (IC, 4 mg in 1 min) injected distal to the occlusion through an over-the-wire balloon before the first balloon dilation	179 min	—	6.0 months
Vijayalakshmi 2006 [30]	UK	101 (51/50)	60.6	79.2	46.5	5.9	28.7	STEMI and NSTEMI	70	NA	NA	Adenosine (IC, 30 µg in 10 mL of heparinised saline) was given very quickly and a repeat angiogram of the relevant vessel was recorded within 10 s	NA	—	6.0 months
Hendler 2006 [31]	Israel	20 (10/10)	NA	NA	NA	NA	NA	STEMI	NA	NA	NA	Adenosine (IC, 60–120 µg) was administered to achieve an activated clotting time between 250–300 s	NA	—	1.0 month
Ji 2007 [32]	China	50 (23/27)	60.0	82.0	54.0	16.0	50.0	STEMI	NA	100.0	NA	The balloon was inflated and then deflated to initiate reperfusion of the ischemic territory, then adenosine (IC, 300 µg) was hand-injected into the vascular bed for 1 min through the guiding catheter into opening of left or right coronary artery	268 min	—	1.0 month
Tian 2008 [33]	China	26 (12/24)	53.1	65.4	NA	NA	NA	STEMI	NA	100.0	0	Adenosine (IC, 2 mg/1 min for 10 min) was given when the guide wire crossed the lesion through PCI, then the balloon was dilated and stent was implanted at the lesion	NA	MACE: sudden death, heart failure, re-infarction, angina pectoris	1.0 month
Stoel 2008 [34]	Netherlands	49 (27/22)	66.9	65.3	40.8	10.2	34.7	STEMI	70	100.0	NA	Adenosine (IC, 60 mg in 5–10 min) infused in 5–10 min	196 min	—	12.0 months
Fokkema 2009 [35]	Netherlands	448 (226/222)	62.4	74.8	37.0	10.2	57.8	STEMI	70	95.8	0–3	Adenosine (IC, 120 µg twice), the first bolus injection was given after thrombus aspiration and the second after stenting of the infarct-related artery	180 min	MACE: mortality, reinfarction, and target vessel revascularization	1.0 month
Grygier 2011 [36]	Poland	70 (35/35)	64.9	62.9	62.9	22.9	50.0	STEMI	70	100.0	0–2	Adenosine (IC, 1 mg for RCA and 2 mg for LCA twice), immediately after crossing the lesion of the infarct-related artery with guidewire and then after the first balloon inflation	270 min	MACE: mortality, recurrent AMI, cardiac arrest, cardiogenic shock, heart failure, and recurrent angina episodes	1.0 month
Desmet 2011 [37]	Belgium	110 (56/54)	61.0	81.8	40.0	10.0	49.1	STEMI	70	100.0	0–3	The balloon was inflated, adenosine (IC, 4 mg bonus) was injected by hand through the central lumen of the balloon catheter into the distal vascular bed over 1 min	215 min	—	4.0 months
Wang 2012 [38]	China	69 (35/34)	56.5	82.6	58.0	18.9	46.4	STEMI	NA	100.0	0–3	15 min prior to the implantation of the stent, adenosine (IV, 50 µg/kg/min for 3 hours) was started for 3 hours	336 min	MACE: recurrent angina, recurrent AMI, heart failure and cardiac death	1.0 month
Zhang 2012 [39]	China	63 (32/31)	64.9	81.0	58.7	28.6	55.6	STEMI	NA	100.0	0–3	Adenosine (IV, 50–70 µg/kg/min for 3 hours), drugs were given to the patients immediately after the guide wire crossed the culprit lesion	297 min	MACE: cardiac death, non-cardiac death, nonfatal AMI, heart failure	1.0 month
Niccoli 2013 [40]	Italy	160 (80/80)	63.5	75.6	56.9	23.1	58.1	STEMI	70	100.0	0–1	Adenosine (IC, 120 µg bolus+2 mg over 2 min) was given distal to the occluded site after thrombus aspiration	279 min	MACE: mortality, AMI, target lesion revascularization, and heart failure	1.0 month
Tong 2013 [41]	China	258 (130/128)	60.9	76.4	43.8	19.0	57.8	STEMI	70	100.0	NA	Adenosine (IC, 2 mg in 1 min twice) was given after thrombus aspiration and the second after stenting of the infarct-related artery	319 min	MACE: mortality, AMI, target vessel revascularization, and NYHA ≥2	1.0 and 12.0 month
Darahim 2014 [42]	Egypt	60 (20/40)	52.7	73.3	41.7	35.0	63.3	STEMI	70	100.0	0–2	The balloon was inflated, adenosine (IC, 6 mg bonus) was hand injected over 30 s into the distal vessel	244 min	No reflow was diagnosed when there was a reduction of 1 or more in the TIMI grade; MACE: nonfatal AMI and mortality	In-hospital
Faruk Akturk 2014 [43]	Turkey	31 (16/15)	57.0	80.6	54.8	29.0	48.4	STEMI	50	NA	NA	Adenosine (IC, 240 µg) was administered in 1 min through the guiding catheter	228 min	—	In-hospital, 6.0 months
Garcia-Dorado 2014 [44]	Spain	197 (100/97)	59.2	86.3	46.7	15.2	51.8	STEMI	70	85.3	0–1	Adenosine (IC, 2.25 mg/min for 2 min) was administered as a 2-minute intracoronary bolus distal to the culprit lesion by means of an intracoronary infusion microcatheter	154 min	—	6.0 months
Naghshtabrizi 2020 [45]	Iran	104 (52/52)	NA	NA	NA	NA	NA	STEMI	NA	NA	NA	Adenosine (IC, 2 bolus, 40 µg/bolus and diluted in 10 mL saline). After crossing the wire through the occlusion site, the first dose of the study drug or placebo was administered by an over-the-wire balloon. After successful stenting, the second dose was administered	NA	No reflow was defined on the basis of TIMI grade flow and ST-segment resolution	1.0 month
Sadeghian 2022 [46]	Iran	228 (110/118)	58.6	79.4	32.5	20.2	40.4	STEMI	NA	100.0	0–1	Adenosine (IC, 200 µg for RCA and 400 µg for LCA) was infused just before stenting	NA	MBG <2 was considered as no-reflow	In-hospital

STEMI, ST-elevation myocardial infarction; DM, diabetes mellitus; MACEs, major adverse cardiovascular event; AMI, acute myocardial infarction; STR, ST resolution; USA, The United States of America; UK, The United Kingdom of Great Britain and Northern Ireland; NA; not applicable; IC, intracoronary; RCA, right coronary artery; LCA, left coronary artery; MBG, myocardial blush grade.

### 3.3 ST Resolution

**Fig. 2.**
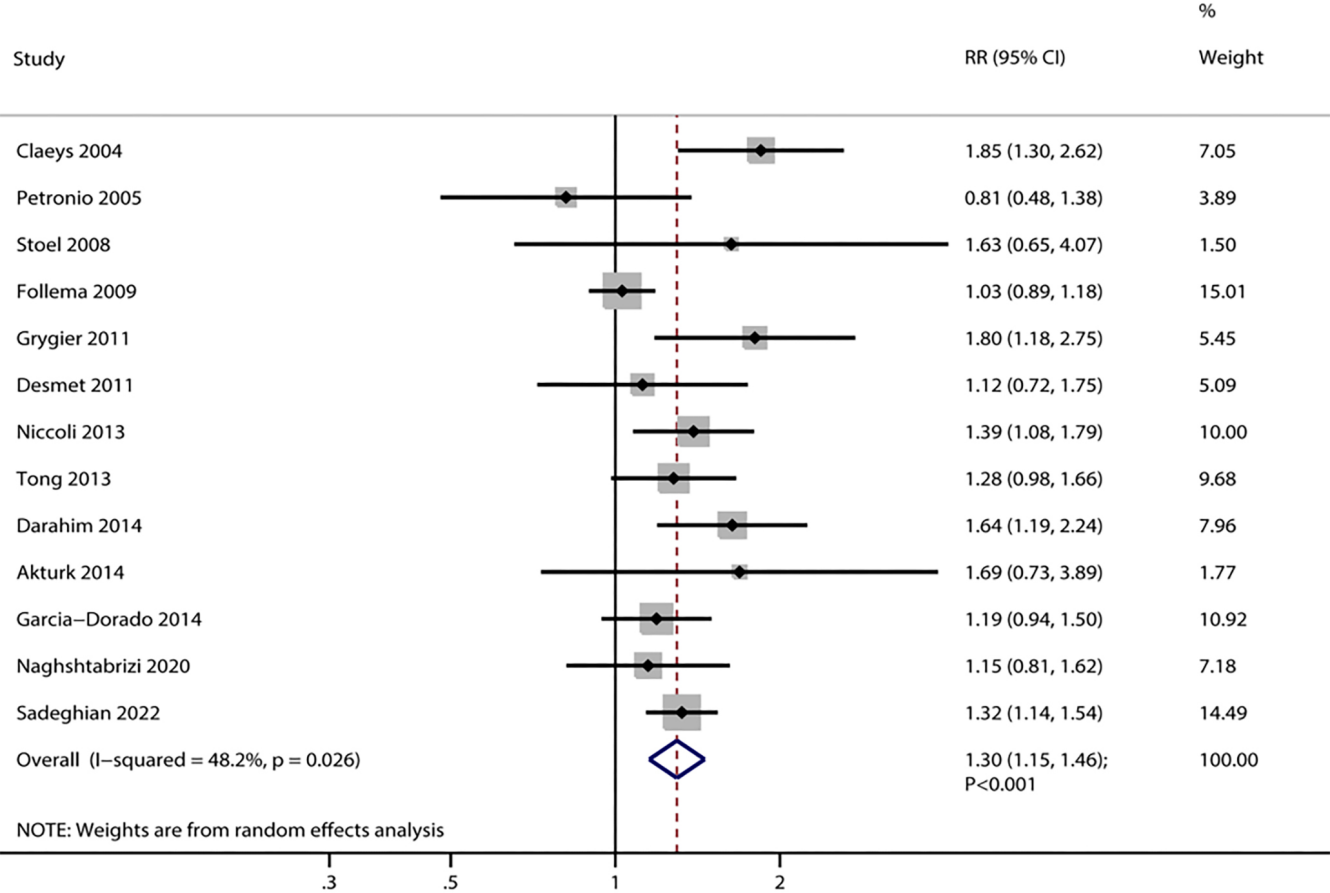
**Effect of adenosine on the incidence of ST resolution**. RR, relative risk; CI, confidence interval.

13 trials reported the effect of adenosine on the incidence of ST resolution. The pooled results indicated that adenosine was associated with an increased incidence of ST resolution (RR, 1.30; 95% CI, 1.15–1.46; *p* < 0.001) (Fig. 2). Notably, considerable heterogeneity was detected among the incorporated trials (*I*^2^ = 48.2%; *p* = 0.026). A sensitivity analysis confirmed the durability of the pooled findings, as it remained unaltered even when each study was sequentially omitted from the analysis (**Supplementary Fig. 1**). A subgroup analysis found a significant difference between adenosine and placebo for the incidence of ST resolution in most subgroups, whereas adenosine was not associated with the incidence of ST resolution if the male proportion was ≥80.0% or ischemic time to therapy was <240 min (Table 2). Moreover, the benefit effect of adenosine on the incidence of ST resolution in ischemic time to therapy ≥240.0 min was greater than ischemic time to therapy <240.0 min (ratio of RR: 1.40; 95% CI, 1.17–1.67; *p* < 0.001).

**Table 2.** **Subgroup analyses for ST resolution and MACE**.

Outcomes	Factors	Subgroups	No. of trials	RR and 95% CI	*p* value	*I*^2^ (%)/Q statistic	Interaction *t* test	Ratio of RR between subgroups
ST resolution	Age (years)	≥60.0	7	1.35 (1.11–1.64)	0.003	63.7/0.011	0.730	1.05 (0.81–1.36)
		<60.0	5	1.29 (1.09–1.53)	0.003	36.1/0.181		
	Male (%)	≥80.0	4	1.14 (0.95–1.38)	0.162	0.0/0.465	0.113	0.82 (0.64–1.05)
		<80.0	8	1.39 (1.18–1.62)	<0.001	64.0/0.007		
	Hypertension (%)	≥50.0	3	1.50 (1.22–1.85)	<0.001	0.0/0.566	0.242	1.16 (0.90–1.50)
		<50.0	8	1.29 (1.12–1.49)	<0.001	56.8/0.023		
	DM (%)	≥20.0	6	1.39 (1.20–1.63)	<0.001	28.7/0.220	0.343	1.12 (0.89–1.42)
		<20.0	6	1.24 (1.04–1.49)	0.018	55.5/0.047		
	Current smoking (%)	≥50.0	7	1.26 (1.07–1.49)	0.006	61.1/0.017	0.360	0.90 (0.72–1.13)
		<50.0	5	1.40 (1.20–1.63)	<0.001	8.8/0.356		
	Route	IC	13	1.30 (1.15–1.46)	<0.001	48.2/0.026	-	-
		IV	-	-	-	-		
	Ischemic time to therapy (min)	≥240.0	5	1.51 (1.31–1.73)	<0.001	2.9/0.390	<0.001	1.40 (1.17–1.67)
		<240.0	6	1.08 (0.96–1.20)	0.200	0.0/0.522		
MACE	Age (years)	≥60.0	8	0.69 (0.50–0.93)	0.017	7.9/0.369	0.625	1.19 (0.59–2.39)
		<60.0	4	0.58 (0.31–1.08)	0.087	0.0/0.811		
	Male (%)	≥80.0	3	0.77 (0.49–1.20)	0.243	0.0/0.693	0.442	1.24 (0.71–2.16)
		<80.0	9	0.62 (0.45–0.86)	0.005	0.0/0.484		
	Hypertension (%)	≥50.0	4	0.63 (0.42–0.95)	0.028	0.0/0.403	0.400	0.79 (0.45–1.37)
		<50.0	6	0.80 (0.55–1.17)	0.251	0.0/0.762		
	DM (%)	≥20.0	4	0.64 (0.42–0.98)	0.042	0.0/0.392	0.488	0.82 (0.47–1.44)
		<20.0	6	0.78 (0.54–1.12)	0.178	0.0/0.738		
	Current smoking (%)	≥50.0	7	0.74 (0.55–0.99)	0.044	0.0/0.448	0.604	1.25 (0.53–2.95)
		<50.0	3	0.59 (0.26–1.30)	0.189	0.0/0.968		
	Route	IC	10	0.63 (0.46–0.85)	0.003	0.0/0.582	0.422	0.78 (0.42–1.44)
		IV	2	0.81 (0.48–1.39)	0.450	0.0/0.448		
	Ischemic time to therapy (min)	≥240.0	7	0.65 (0.47–0.90)	0.009	0.0/0.788	0.741	0.89 (0.45–1.77)
		<240.0	4	0.73 (0.40–1.35)	0.315	34.1/0.208		

RR, relative risk; CI, confidence interval; DM, diabetes mellitus; MACE, major adverse cardiovascular event; I^2^, inconsistency index; IC, intracoronary; IV, intravenous; TIMI, Thrombolysis in Myocardial Infarction; IV, intravenous; NSTEMI, non-ST-elevation myocardial infarction; PCI, percutaneous coronary intervention; NYHA, New York Heart Association.

### 3.4 Major Adverse Cardiovascular Events

**Fig. 3.**
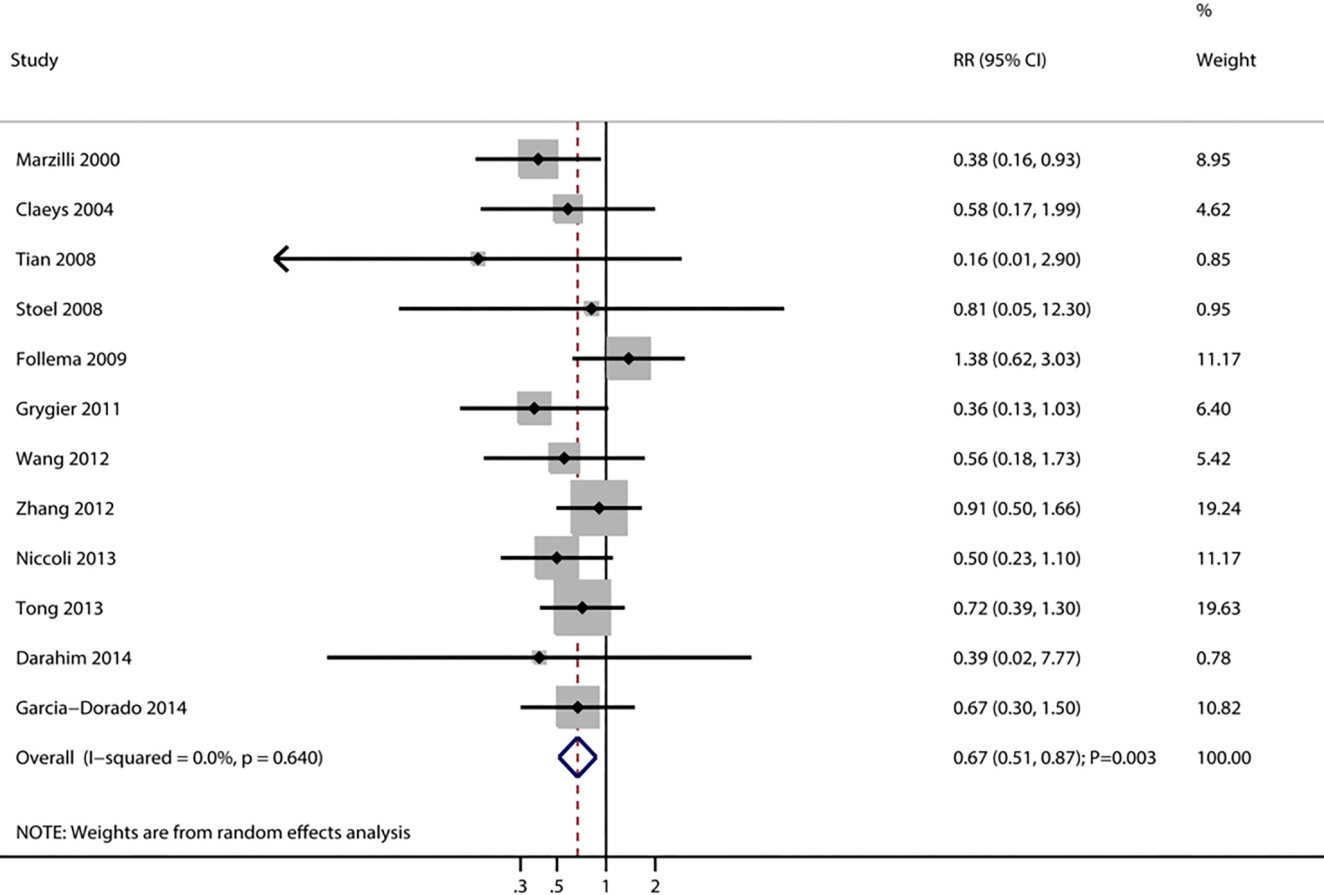
**Effect of adenosine on the risk of major adverse cardiovascular events**. RR, relative risk; CI, confidence interval.

12 trials reported the effect of adenosine on the risk of MACEs. The summary RR indicated that adenosine significantly reduced the risk of MACEs (RR, 0.67; 95% CI, 0.51–0.87; *p* = 0.003) (Fig. 3), and no evidence of heterogeneity was observed (*I*^2^ = 0.0%; *p* = 0.640). The sensitivity analysis demonstrated that the aggregated conclusion was unaffected by the stepwise exclusion of any individual study (**Supplementary Fig. 2**). The subgroup analysis revealed that adenosine was associated with a lower risk of MACEs when age was 60.0 years or older, the male proportion was <80.0%, the hypertension proportion was ≥50.0%, the DM proportion was ≥20.0%, the current proportion of smokers was ≥50.0%, the administration of adenosine was intracoronary, and the ischemic time to therapy was ≥240.0 min (Table 2).

### 3.5 No Reflow, and MBG 0 to 1

There were five and seven trials available for no reflow and MBG 0 to 1, respectively (**Supplementary Fig. 3**). Adenosine was associated with a reduced risk of no reflow (RR, 0.35; 95% CI, 0.24–0.52; *p* < 0.001) and MBG 0 to 1 (RR, 0.75; 95% CI, 0.58–0.99; *p* = 0.041). There was no significant heterogeneity for no reflow (*I*^2^ = 0.0%; *p* = 0.843) and MBG 0 to 1 (*I*^2^ = 36.8%; *p* = 0.148).

### 3.6 All-Cause Mortality, Cardiac Death, Thrombosis, Reinfarction, and Heart Failure

There were 12, 5, 10, 7, and 9 available trials for all-cause mortality, cardiac death, thrombosis, reinfarction, and heart failure, respectively (**Supplementary Fig. 4**). Adenosine was associated with a reduced risk of heart failure (RR, 0.66; 95% CI, 0.44–0.99; *p* = 0.044); however, it had no significant effects on the risk of all-cause mortality (RR, 0.73; 95% CI, 0.41–1.28; *p* = 0.272), cardiac death (RR, 0.65; 95% CI, 0.28–1.55; *p* = 0.335), thrombosis (RR, 0.64; 95% CI, 0.41–1.00; *p* = 0.052), or reinfarction (RR, 0.82; 95% CI, 0.36–1.90; *p* = 0.648). There was no significant heterogeneity for all-cause mortality (*I*^2^ = 0.0%; *p* = 0.825), cardiac death (*I*^2^ = 0.0%; *p* = 0.477), thrombosis (*I*^2^ = 35.8%; *p* = 0.122), reinfarction (*I*^2^ = 0.0%; *p* = 0.885), or heart failure (*I*^2^ = 0.0%; *p* = 0.888).

### 3.7 Adverse Events

Eight, six, five, and three trials were available for advanced AV blocks, hypotension, VT/VF, and bradycardia, respectively (**Supplementary Fig. 5**). Adenosine was associated with an increased risk of advanced AV block (RR, 5.83; 95% CI, 3.38–10.05; *p* < 0.001) and hypotension (RR, 2.77; 95% CI, 1.24–6.15; *p* = 0.013). However, there were no significant differences between adenosine and placebo in terms of the risks of VT/VF (RR, 0.83; 95% CI, 0.35–1.96; *p* = 0.665) and bradycardia (RR, 2.96; 95% CI, 0.43–20.41; *p* = 0.272). We noted potentially significant heterogeneity for hypotension (*I*^2^ = 58.9%; *p* = 0.032) and bradycardia (*I*^2^ = 80.3%; *p* = 0.006); however, there was no evidence of heterogeneity for advanced AV blocks (*I*^2^ = 0.0%; *p* = 0.511) and VT/VF (*I*^2^ = 0.0%; *p* = 0.758).

### 3.8 CK-MB Peek Value and LVEF

10 and 13 trials were available for the CK-MB peak value and LVEF, respectively. Adenosine was associated with a lower CK-MB peak value (WMD: –36.94; 95% CI, –73.76 to –0.11; *p* = 0.049) (**Supplementary Fig. 6**); however, it had no significant effect on the LVEF level (WMD: 2.16; 95% CI, –0.01 to 4.33; *p* = 0.051) (**Supplementary Fig. 7**). Significant heterogeneity was observed in the CK-MB peak value (*I*^2^ = 78.9%; *p* < 0.001) and LVEF (*I*^2^ = 74.5%; *p* < 0.001).

### 3.9 Publication Bias

**Fig. 4.**
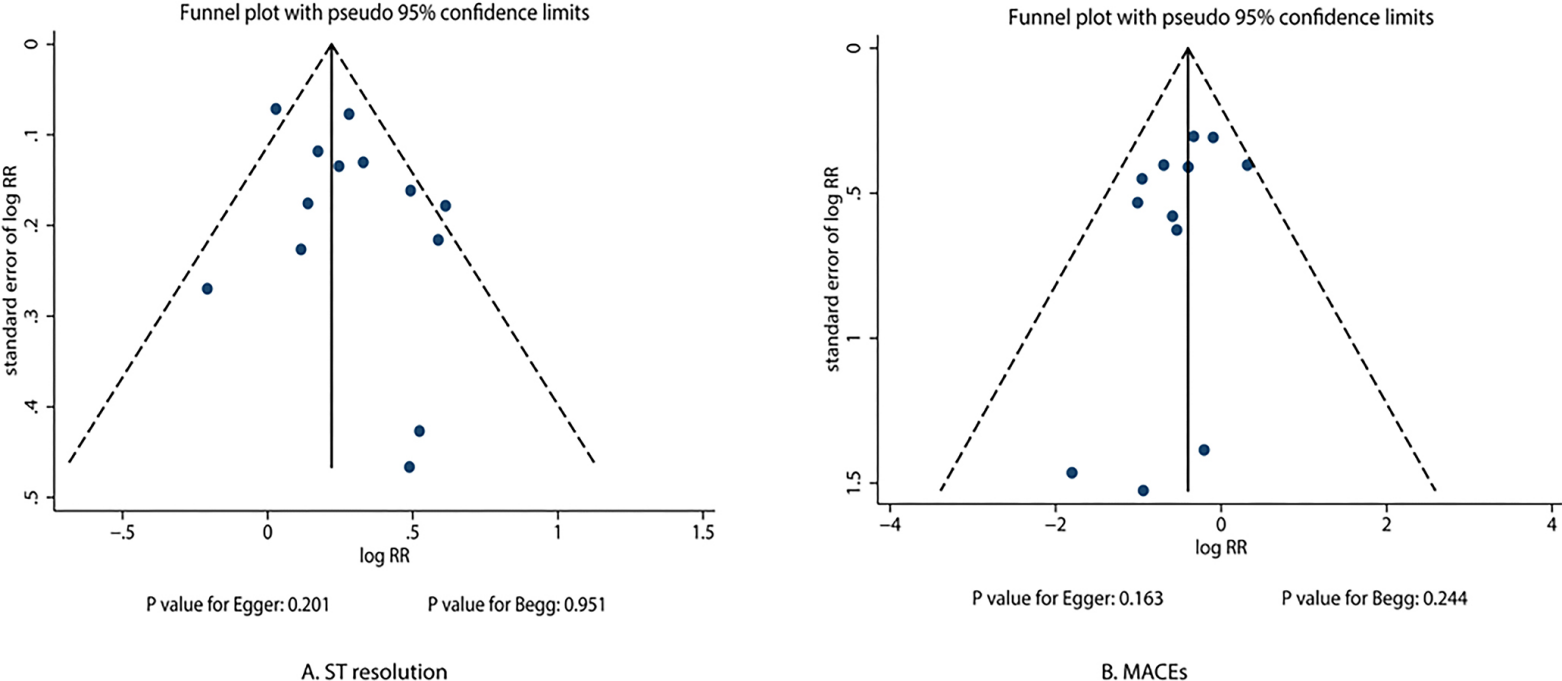
**Funnel plots for ST resolution and major adverse cardiovascular events**. RR, relative risk; MACEs, major adverse cardiovascular events.

There was no significant publication bias for ST resolution (*p*_𝐸𝑔𝑔𝑒𝑟_ = 0.201; *p*_𝐵𝑒𝑔𝑔_ = 0.951) and MACEs (*p*_𝐸𝑔𝑔𝑒𝑟_ = 0.163; *p*_𝐵𝑒𝑔𝑔_ = 0.244) (Fig. 4).

## 4. Discussion

The current study was based on RCTs to evaluate the impact of adenosine on both angiographic and clinical outcomes in patients with AMI undergoing PCI. An aggregate of 2467 AMI patients was included, showcasing a diverse spectrum of patient characteristics. Adenosine for AMI patients undergoing PCI was able to improve ST resolution, no reflow, MBG 0 to 1, MACEs, heart failure, and CK-MB peak values. Although the use of adenosine showed a protective trend for the risk of all-cause mortality, cardiac death, thrombosis, reinfarction, and LVEF, the differences between adenosine and control groups were not statistically significant. Furthermore, the risks of VT/VF and bradycardia for patients treated with adenosine were not affected.

Several systematic reviews have been conducted to examine the effects of adenosine [47, 48, 49, 50]. Singh *et al*. [47] identified trials published until May 2011 and seven RCTs were identified, and they found that intracoronary administration of adenosine was well-tolerated and resulted in improved electrocardiographic outcomes. Moreover, intracoronary administration of adenosine provides protection against the risks of MACEs, heart failure, and cardiac death. Navarese *et al*. [48] identified trials published until August 2011 and 10 RCTs were included, they reported that the use of adenosine was associated with a reduced risk of no reflow. A total of 15 RCTs were identified for trials published until December 2014 during a study by Gao *et al*. [49], who indicated that adenosine could protect against the risks of heart failure, no reflow, and MBG 0 to 1. Polimeni *et al*. [50] identified 13 RCTs published until February 2015 and suggested that intracoronary administration of adenosine was associated with an increased incidence of ST resolution and a lower risk of MACEs for STEMI patients undergoing primary PCI. However, several RCTs have already been published, thus the results should be updated for the therapeutic effects of adenosine. Moreover, whether the effects of adenosine treatment are affected by patient characteristics remains controversial. Therefore, this systematic review and meta-analysis aimed to update the effects of adenosine on patients with AMI undergoing PCI.

The results indicated that the use of adenosine could significantly improve ST resolution, no reflow, and MBG 0 to 1. The potential reason for this could be that adenosine activates 4 receptors, which could dilate the coronary vessels and attenuate reperfusion injury by decreasing neutrophil-mediated mechanical obstruction of the capillary channels. Our study found that adenosine significantly reduced the risks of MACEs and heart failure; although the risks of all-cause mortality, cardiac death, thrombosis, and reinfarction after treatment with adenosine were not affected, a protective trend was observed for patients receiving adenosine, which could be explained by the lower incidence of these clinical outcomes. Considering the various definitions of MACEs across the included trials, the prevalence of MACEs was relatively high, and the power was sufficient to detect potential significant differences. Moreover, the beneficial effect of adenosine could be attributed to the reduced infarct size and improved cardiac function [51, 52, 53] including: (1) adenosine can reduce the occurrence of arrhythmias by regulating the electrophysiological activity within cardiac cells, which is particularly important for post-PCI patients; (2) adenosine can dilate coronary arteries and other blood vessels, increase blood supply to the heart, improve myocardial ischemia, and help reduce myocardial damage after PCI surgery; (3) adenosine has anti-inflammatory and antioxidant effects, which can alleviate the inflammatory response and oxidative stress caused by PCI surgery, helping to protect cardiac tissues; and (4) adenosine can protect myocardium from ischemia-reperfusion injury by regulating the metabolism and function of myocardial cells, thus helping to maintain cardiac function.

The results of this study showed that the use of adenosine significantly increased the risk of advanced AV block and hypotension. However, most adverse events were transient because of the short half-life of adenosine, which did not cause clinical sequelae. The potential reason for these results could be explained by the fact that adenosine can slow the heart rate by acting on the sinoatrial node and AV node of the heart, prolonging atrioventricular conduction time, sometimes leading to AV block. In addition, adenosine can dilate blood vessels, causing a decrease in blood pressure, so hypotension may occur when using adenosine. Moreover, adenosine was associated with a lower CK-MB peak value and might increase LVEF rates, which are significantly related to infarct size and subsequent clinical outcomes.

Stratified analyses of ST resolution and MACEs were also performed, and the effect of adenosine on the incidence of ST resolution could be affected by ischemic time to therapy. Moreover, the risk of MACEs was reduced for patients treated with adenosine when age was 60.0 years or older, the male proportion was <80.0%, the hypertension proportion was ≥50.0%, the DM proportion ≥20.0%, the proportion of current smokers was ≥50.0%, the administration of adenosine was intracoronary, and the ischemic time to therapy was ≥240.0 min. Prolonged myocardial ischemia exacerbates metabolic waste accumulation and oxidative stress, leading to severe reperfusion injury. Adenosine, through its antioxidative, anti-inflammatory, and calcium channel modulation properties, effectively alleviates such injuries, particularly benefiting patients with prolonged ischemia [54]. Moreover, extended ischemia aggravates microcirculatory damage, impeding blood flow restoration. By dilating microvessels, adenosine is pivotal in reinstating microcirculatory blood flow in long-ischemic myocardium, thereby aiding in salvaging more critically endangered myocardial cells [55]. These results suggest that adenosine should be administered to high-risk AMI patients to achieve better treatment effects.

Several limitations of this study should be acknowledged. Although all of the included studies were designed as RCTs, the methodological quality varied, and the recommendations of the results were restricted. Variations existed in the dosage of adenosine administered across the different trials included, potentially influencing the outcome for AMI patients undergoing PCI. The dosage of adenosine requires meticulous control to ensure optimal therapeutic efficacy while avoiding adverse reactions. Lower doses may be insufficient to adequately dilate both coronary arteries and microvessels, failing to effectively improve myocardial blood flow and thereby compromising treatment outcomes. Besides, the duration of adenosine therapy is equally crucial, as it pertains to the sustained action of the drug in the body and the ongoing myocardial protective effects. Short-term use may only temporarily enhance blood flow without fully realizing the long-term benefits of its anti-inflammatory, antioxidative, and myocardial repair-promoting properties. The severity of AMI differed among the included trials, and the treatment effects of adenosine might have been affected by the disease status. The definition of ST resolution differed, which could have affected the net effect of adenosine on the incidence of ST resolution. Furthermore, the timeframe of the investigated outcomes differed across the included trials, which could affect the therapeutic effects of adenosine. The analysis relies on published literature, thus publication bias is an inevitable issue. Finally, the detailed analyses were restricted because the analysis was based on pooled data.

## 5. Conclusions

Adenosine was superior to placebo for improving ST resolution, MACEs, no reflow, MBG 0 to 1, heart failure, and CK-MB peak value. Moreover, although adenosine significantly increased the risk of advanced AV block and hypotension, these events were transient and did not cause clinical sequelae. Further large-scale RCTs ought to be conducted to investigate the long-term effects of different adenosine administrations in patients suffering from AMI who are undergoing PCI.

## Data Availability

The datasets used and/or analyzed during the current study are available from the corresponding author on reasonable request.

## Supplementary Material

Supplementary material associated with this article can be found, in the online version, at https://doi.org/10.31083/RCM24065.
