# Supplementary material for: Adenosine as an Adjunctive Therapy for Acute Myocardial Infarction Undergoing Primary Percutaneous Coronary Intervention: A Systematic Review and Meta-Analysis of Randomized Controlled Trials
Source: Rev Cardiovasc Med. 2025 Feb 12;26(2):24065. doi: 10.31083/RCM24065 (PMC11868911; doi:10.31083/RCM24065)
Supplement: Supplementary file 1 [file 2153-8174-26-2-24065-s1.zip › RCM24065-Supplementary Material-V2.docx]

Supplementary Table 1. Quality assessment of included trials

| Study | Random sequence generation | Allocation concealment | Blinding of participants and personnel | Blinding of outcome assessment | Incomplete outcome data | Selective reporting | Other bias |
| --- | --- | --- | --- | --- | --- | --- | --- |
| Marzilli 2000 | Unclear | Unclear | Low | Low | Low | Low | Low |
| Claeys 2004 | Unclear | Unclear | Low | Low | Low | Low | Low |
| Micari 2005 | Unclear | Unclear | Low | Low | Low | Low | Low |
| Petronio 2005 | High | Unclear | Low | Low | Unclear | Unclear | Unclear |
| Vijayalakshmi 2006 | Low | Unclear | Unclear | Low | Low | Low | Low |
| Hendler 2006 | Unclear | Unclear | Unclear | Low | Unclear | Unclear | Unclear |
| Ji 2007 | Unclear | Unclear | Unclear | Low | Low | Low | Low |
| Tian 2008 | Low | Unclear | Unclear | Low | Low | Low | Unclear |
| Stoel 2008 | Unclear | Unclear | Low | Low | High | Low | Unclear |
| Follema 2009 | Unclear | High | Low | Low | Unclear | Low | Low |
| Grygier 2011 | Unclear | Unclear | Low | Low | Low | Low | Low |
| Desmet 2011 | Low | Low | Low | Low | Low | Low | Low |
| Wang 2012 | Low | Unclear | Unclear | Low | Low | Low | Low |
| Zhang 2012 | Unclear | Unclear | Unclear | Low | Low | Low | Unclear |
| Niccoli 2013 | Low | High | High | Low | Low | Low | Low |
| Tong 2013 | Low | Unclear | Unclear | Low | Low | Low | Low |
| Darahim 2014 | Unclear | Unclear | Low | Low | Low | Low | Unclear |
| Akturk 2014 | Unclear | Unclear | Unclear | Low | Low | Unclear | Unclear |
| Garcia-Dorado 2014 | Unclear | Unclear | Low | Low | Unclear | Low | Low |
| Naghshtabrizi 2020 | Unclear | Unclear | Low | Unclear | Unclear | Unclear | Unclear |
| Sadeghian 2022 | Low | Unclear | Unclear | Low | Low | Low | Unclear |


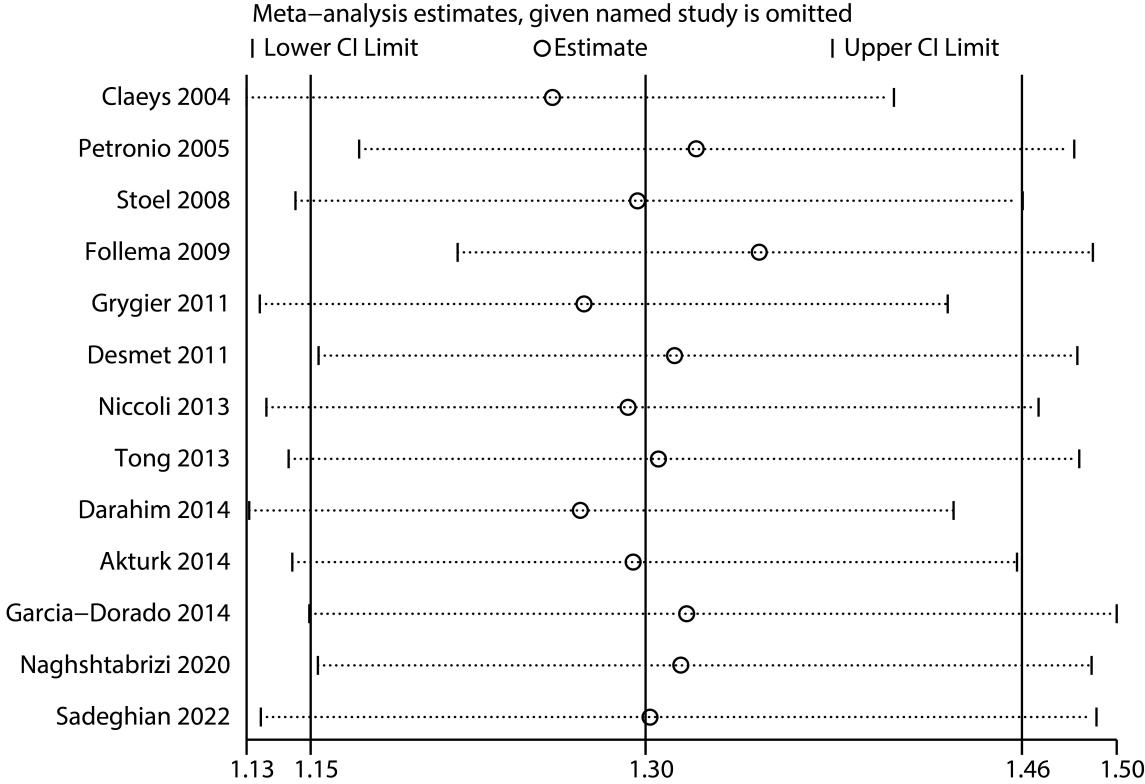


Supplementary Fig. 1. Sensitivity analysis for ST resolution


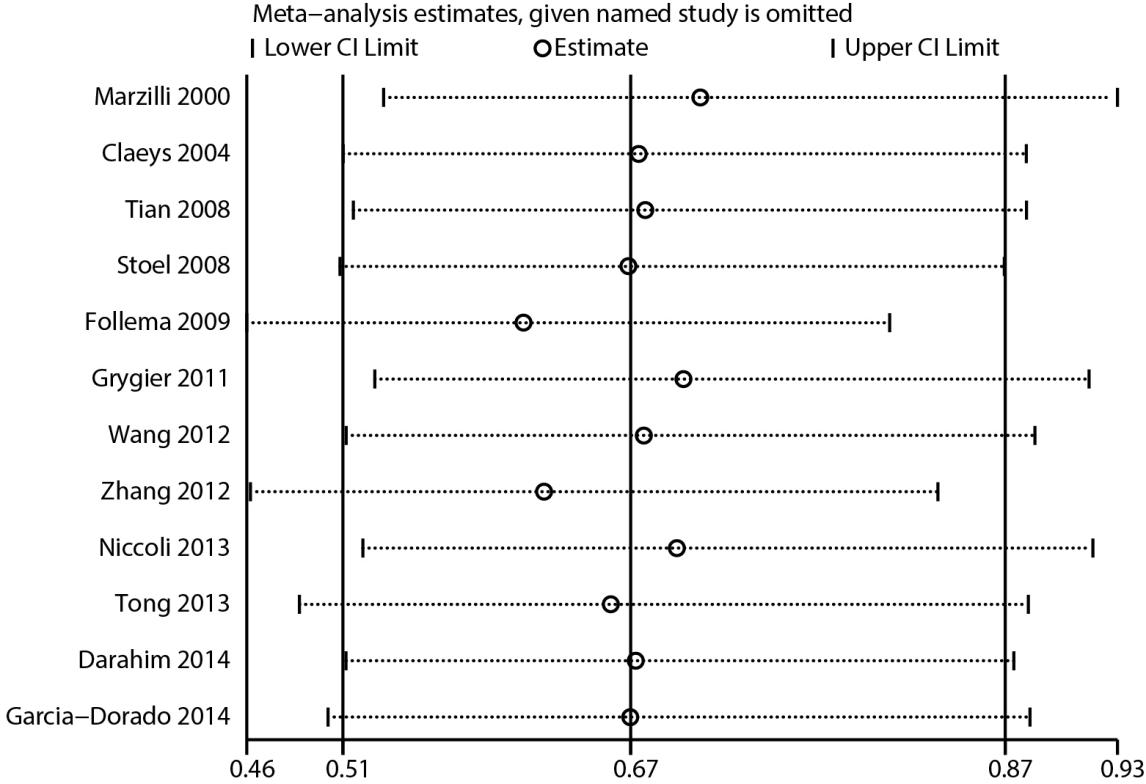


Supplementary Fig. 2. Sensitivity analysis for MACE


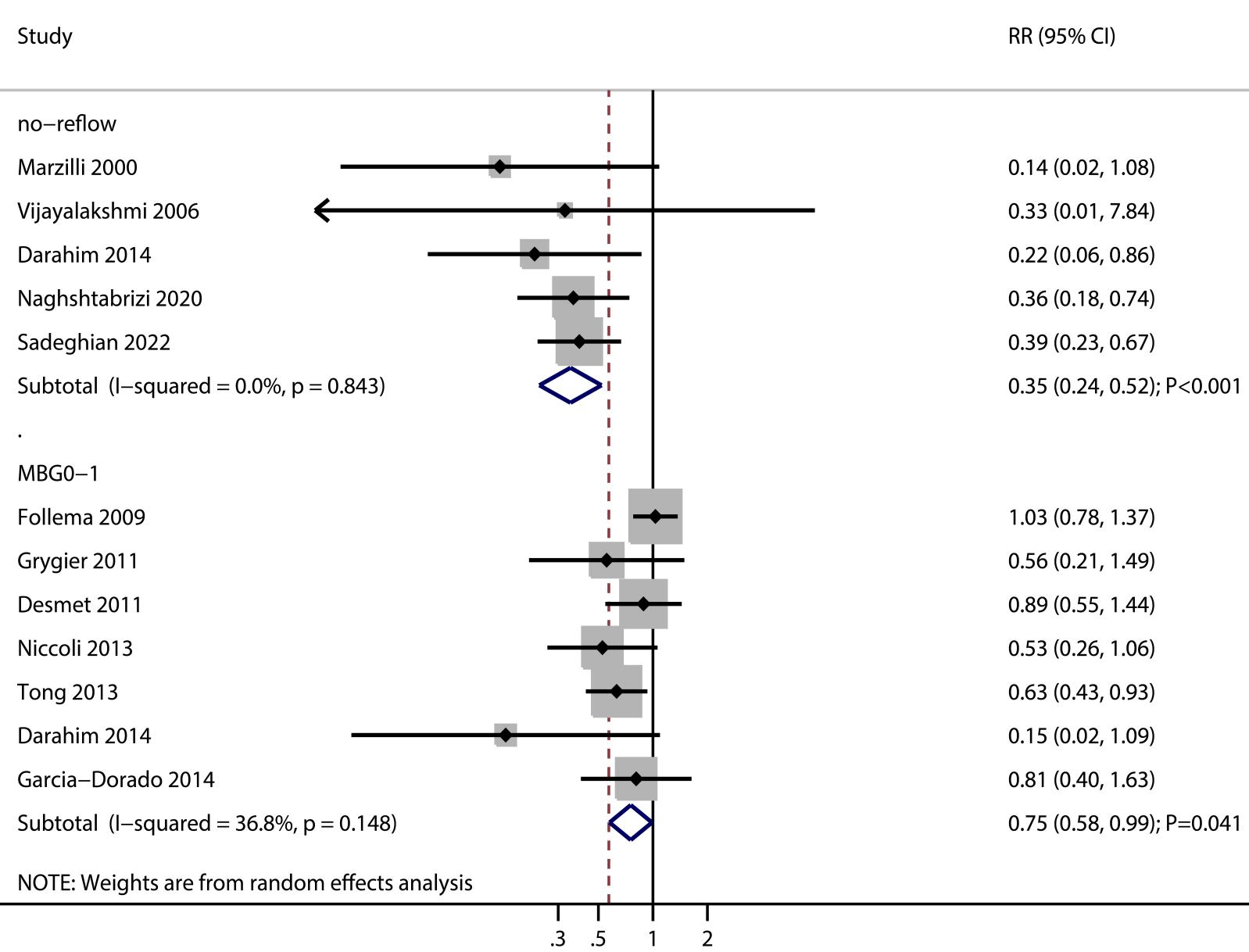


Supplementary Fig. 3. Effect of adenosine on the incidence of no reflow and myocardial blush grade (MBG) 0 to 1. RR: relative risk; CI: confidence interval.


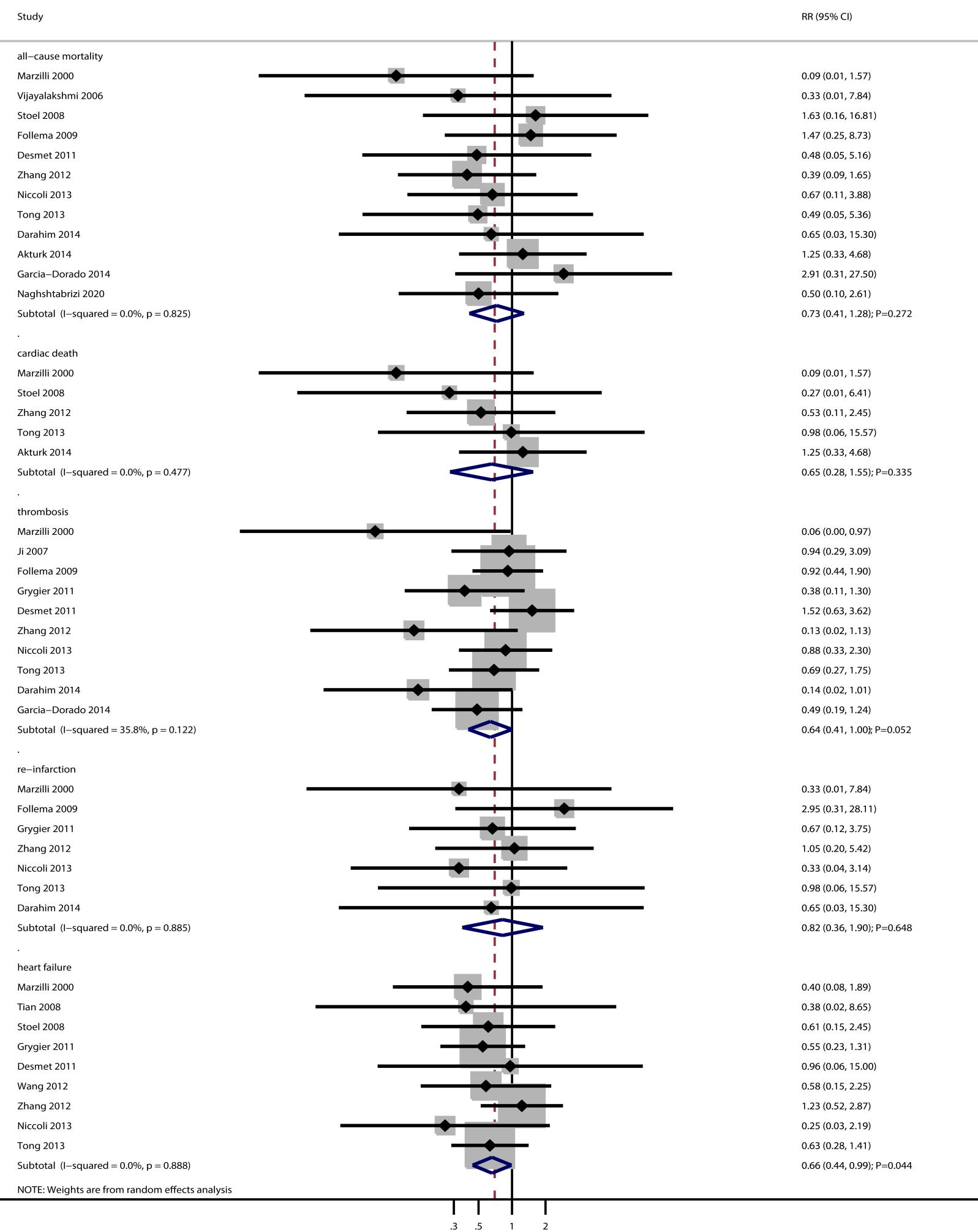


Supplementary Fig. 4. Effect of adenosine on the risks of all-cause mortality, cardiac death, thrombosis, reinfarction, and heart failure


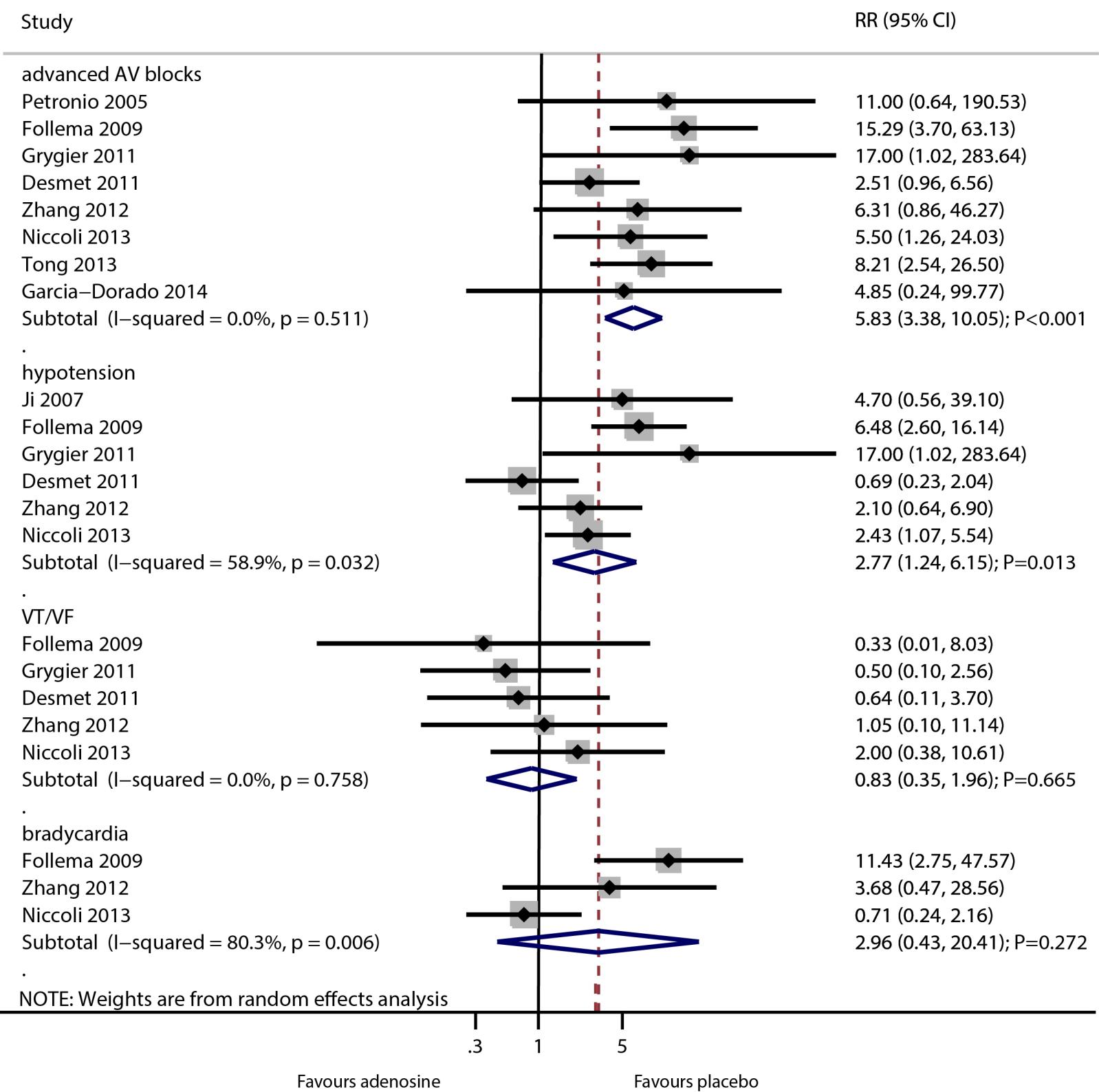


Supplementary Fig. 5. Effect of adenosine on the risks of advanced atrioventricular (AV) blocks, hypotension, ventricular tachycardia (VT)/ventricular fibrillation (VF), and bradycardia


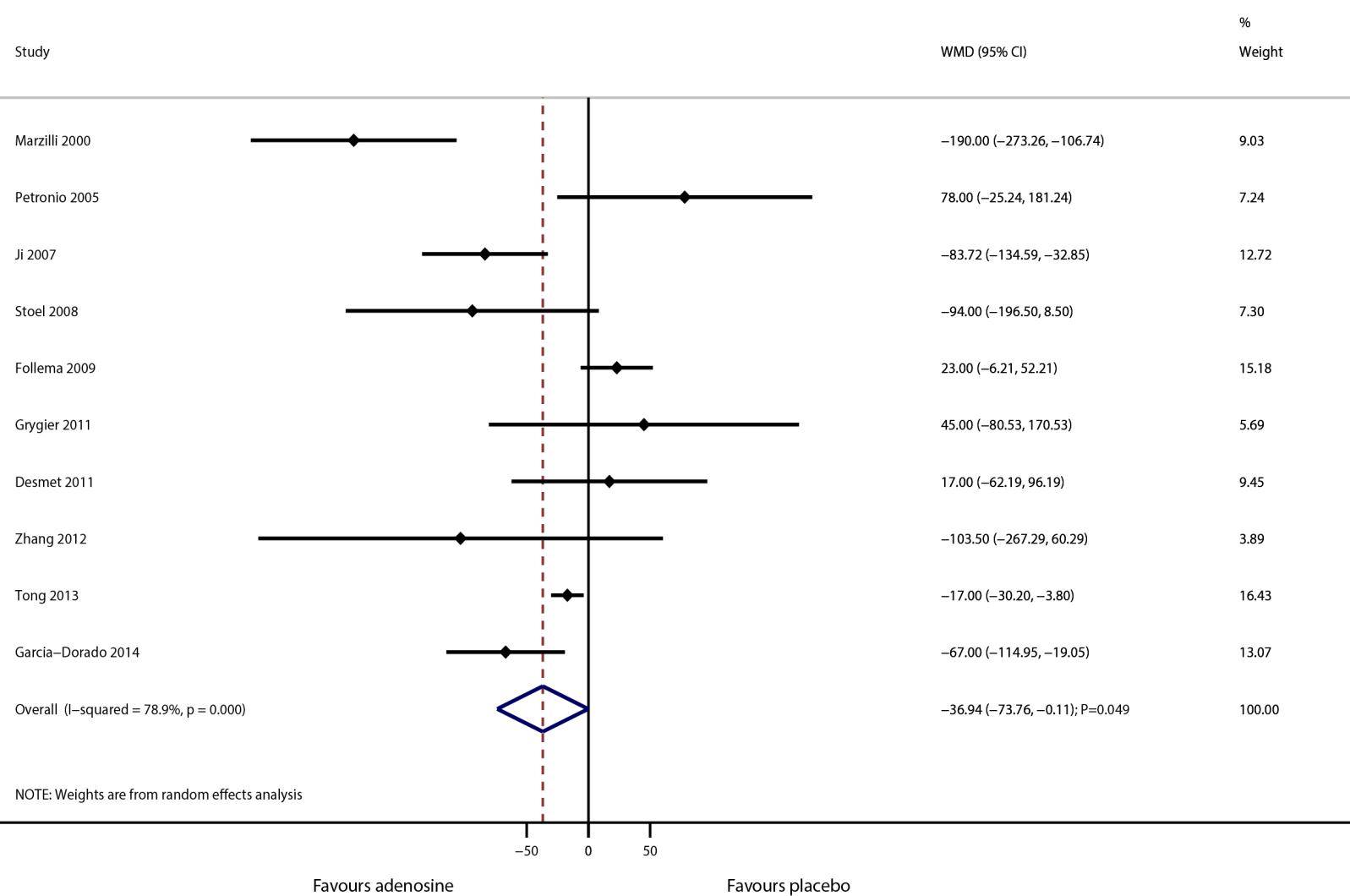


Supplementary Fig. 6. Effect of adenosine on the creatine kinase-MB (CK-MB) peak value


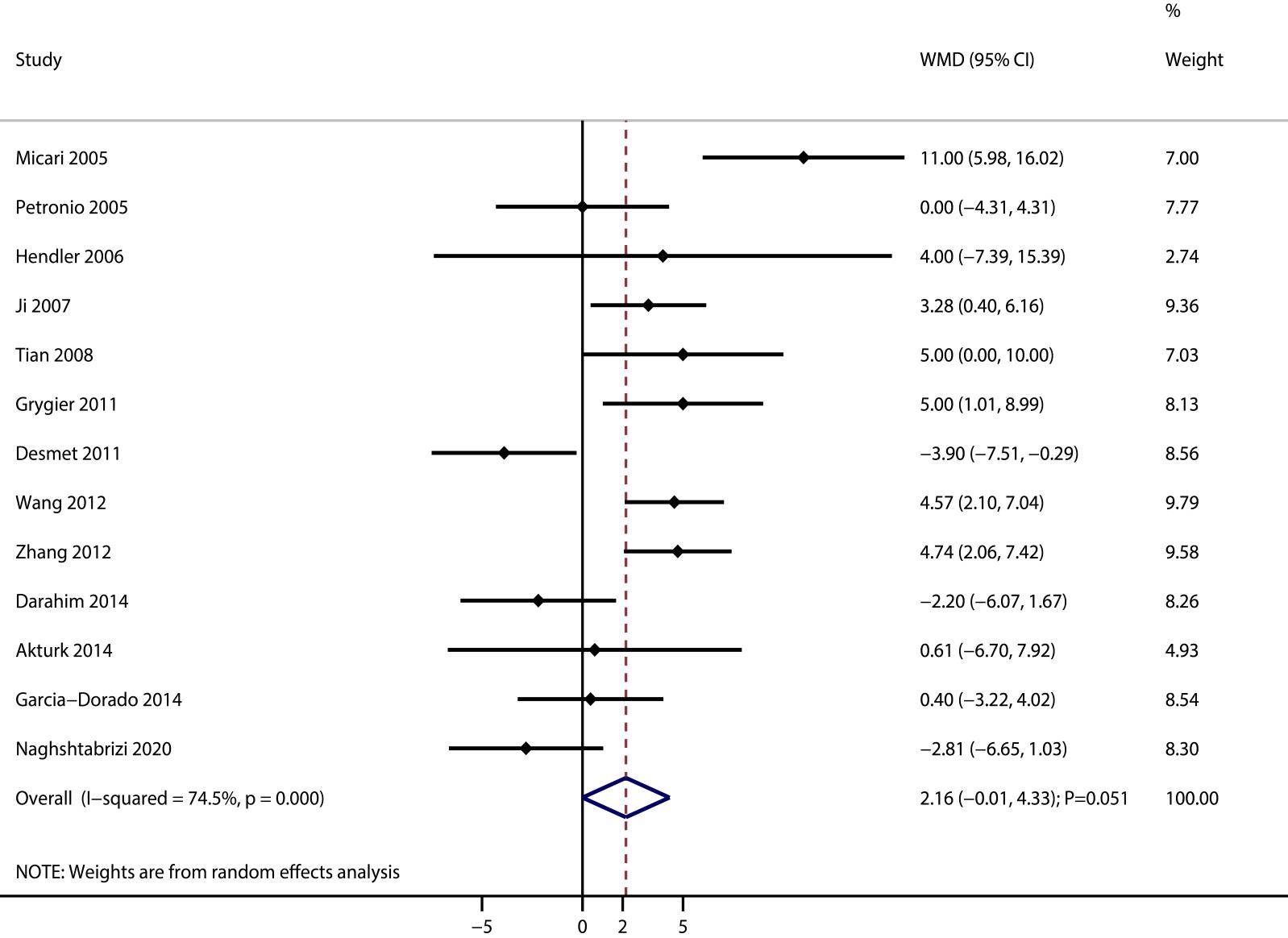


Supplementary Fig. 7. Effect of adenosine on the left ventricular ejection fraction (LVEF)
